# Cardiovascular outcomes with icosapent ethyl by burden of standard modifiable cardiovascular risk factors in REDUCE-IT^☆^

**DOI:** 10.1016/j.ajpc.2026.101458

**Published:** 2026-02-02

**Authors:** Rahul Aggarwal, Deepak L. Bhatt, Michael Miller, Christie M. Ballantyne, Eliot A. Brinton, Terry A. Jacobson, Steven B. Ketchum, Jean-Claude Tardif, Matthew J. Budoff, Gemma A. Figtree, Ph․ Gabriel Steg, REDUCE-IT Investigators

**Affiliations:** a Brigham and Women’s Hospital Heart and Vascular Center, Harvard Medical School, Boston, MA, USA; b Mount Sinai Fuster Heart Hospital, Icahn School of Medicine at Mount Sinai, New York, NY, USA; c Department of Medicine, Crescenz Veterans Affairs Medical Center and University of Pennsylvania School of Medicine, Philadelphia, PA, USA; d Department of Medicine, Baylor College of Medicine, and the Texas Heart Institute, Houston, TX, USA; e Utah Lipid Center, Salt Lake City, UT, USA; f Lipid Clinic and Cardiovascular Risk Reduction Program, Department of Medicine, Emory University School of Medicine, Atlanta, GA, USA; g Amarin Pharma, Inc., Bridgewater, NJ, USA; h Montreal Heart Institute, Université de Montréal, Montreal, Canada; i Lundquist Institute, Harbor-UCLA Medical Center, Torrance, CA, USA; j The University of Sydney, Sydney, NSW, Australia; k Université Paris-Cité, INSERM-UMR1148, Assistance Publique-Hôpitaux de Paris, Hôpital Bichat, French Alliance for Cardiovascular Trials, Paris, France

**Keywords:** Icosapent ethyl, Cardiovascular, Standard modifiable cardiovascular risk factors

**Figure:**
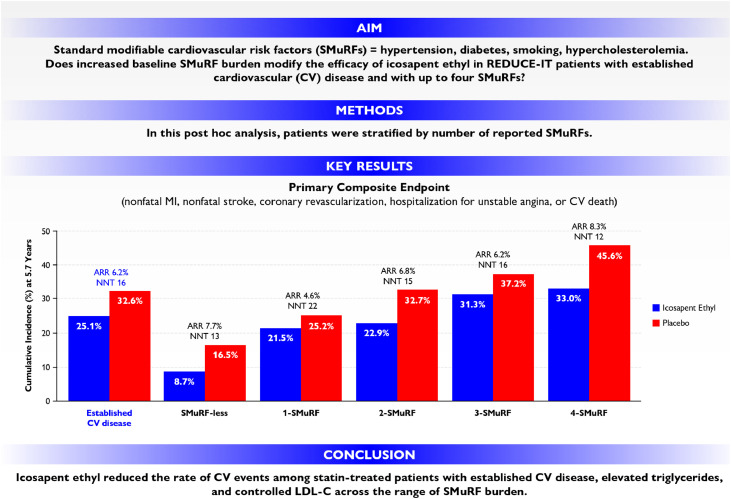
Central Illustration. In the placebo group, increasing SMuRF burden was associated with higher event rates of the primary endpoint. Treatment with icosapent ethyl reduced the rate of cardiovascular events across the range of SMuRF burden.

## 1 Introduction

Coronary artery disease (CAD) remains a leading cause of cardiovascular (CV) mortality. Standard modifiable cardiovascular risk factors (SMuRFs), which include hypertension, hypercholesterolemia, diabetes, and smoking, are well-established risk enhancers for CAD [1].

Icosapent ethyl is a purified ethyl ester of eicosapentaenoic acid (EPA) [2]. In the Reduction of Cardiovascular Events with Icosapent Ethyl–Intervention Trial (REDUCE-IT), icosapent ethyl demonstrated significant reductions in CV events among patients with high CV risk, elevated triglycerides, and well-controlled low-density lipoprotein cholesterol (LDL-C) on background statin therapy [2].

In this analysis of REDUCE-IT, we evaluated whether increased baseline SMuRF burden modified the efficacy of icosapent ethyl among patients with one, two, three, or four SMuRFs and among SMuRF-less patients.

## 2 Methods

The design of REDUCE-IT has previously been described [2,3]. In brief, REDUCE-IT was a randomized, double-blind, placebo-controlled trial that compared icosapent ethyl (2 g twice per day) with placebo. Randomization was stratified by CV risk, ezetimibe use, and geographic region. Patients who were 45 years or older with established CV disease (CVD) or 50 years or older with diabetes and at least one CV risk factor were included. Other inclusion criteria included stable statin therapy, LDL-C of 41 to 100 mg/dL, and fasting triglycerides of 150–499 mg/dL. Inclusion and exclusion criteria have been published [2]. Written informed consent was obtained from all patients in REDUCE-IT, and the protocol was approved by the relevant health authorities, institutional review boards, and ethics committees.

In this post hoc analysis, only patients with established CVD were included, and they were stratified by the number of SMuRFs as reported by the investigators in the medical history case report forms. The primary endpoint was a composite of nonfatal myocardial infarction (MI), nonfatal stroke, coronary revascularization, hospitalization for unstable angina, or CV death. The key secondary endpoint was a composite of nonfatal MI, nonfatal stroke, or CV death.

Differences in endpoints between patients randomized to icosapent ethyl or placebo were analyzed using the Kaplan-Meier method and compared using the log-rank test. Hazard ratios (HRs) and 95 % confidence intervals (CIs) were estimated using a Cox proportional hazards model to compare the risk of endpoints with icosapent ethyl vs placebo. The model was stratified by the 2 randomization factors of geographic region and baseline ezetimibe use. All tests were based on a 2-sided 5 % significance level.

## 3 Results

REDUCE-IT included 8179 patients, with 5785 having established CVD. Among these patients, 5782 (99.9 %) had data on SMuRFs. The SMuRF burden was 131 (2.3 %) patients who were SMuRF-less, 1303 (22.5 %) patients with one SMuRF, 2380 (41.2 %) with two, 1642 (28.4 %) with three, and 326 (5.6 %) with four SMuRFs. At baseline for SMuRF-less patients vs patients with ≥1 SMuRF, the median age was 60.0 vs 64.0 years (*P* < 0.0001), female proportion was 15.3 % vs 21.7 % (*P* = 0.07), and BMI was 27.7 vs 30.4 kg/m^2^ (*P* < 0.0001). Patients with ≥1 SMuRF had history of hypertension (85.6 %), hypercholesterolemia (22.5 %), type 2 diabetes (41.8 %), and were current or former smokers (66.9 %), as reported by the investigator. At baseline, medications for SMURF-less patients vs patients with ≥1 SMuRFs were fewer anti-diabetics (0.8 % vs 36.2 %, *P* < 0.0001), ACE inhibitors or ARBs (45.0 % vs 77.5 %, *P* < 0.0001), beta-blockers (67.9 % vs 80.3 %, *P* = 0.0004), and other anti-hypertensives (80.2 % vs 96.7 %, *P* < 0.0001).

In the placebo group, increasing SMuRF burden was associated with higher event rates of the primary endpoint, with Kaplan Meier cumulative incidences at 5.7 years of 16.5 % for patients who were SMuRF-less, 25.2 % with one, 32.7 % with two, 37.2 % with three, and 45.6 % with four SMuRFs (**Central Illustration**). In the secondary prevention population (i.e., 5785 with established CVD), the Kaplan Meier incidence was 32.6 % for the placebo group and 25.1 % for the IPE group, indicating a significantly lower event rate with icosapent ethyl (hazard ratio [HR]: 0.73 [95 % CI: 0.65, 0.81]; absolute risk reduction [ARR]: 6.2 %; number needed to treat [NNT]: 16; *P* < 0.0001). These findings were consistent by SMuRF burden: SMuRF-less (HR: 0.40 [95 % CI: 0.13, 1.30]), one SMuRF (HR: 0.74 [95 % CI: 0.57, 0.96]), two (HR: 0.69 [95 % CI: 0.58, 0.83]), three (HR: 0.77 [95 % CI: 0.64, 0.93]), and four (HR: 0.74 [95 % CI: 0.50, 1.10]) (P_interaction_=0.83).

Similarly, icosapent ethyl reduced the key secondary endpoint compared with placebo in the secondary prevention population (HR: 0.72 [95 % CI: 0.63, 0.82]; ARR: 4.4 %; NNT: 23; *P* < 0.0001), and by SMuRF burden, including SMuRF-less (HR: 0.37 [95 % CI: 0.07, 1.90]), one SMuRF (HR: 0.80 [95 % CI: 0.58, 1.10]), two (HR: 0.66 [95 % CI: 0.53, 0.82]), three (HR: 0.77 [95 % CI: 0.62, 0.97]), and four (HR: 0.65 [95 % CI: 0.41, 1.04]) (P_interaction_=0.73).

The proportion of patients with at least one treatment-emergent adverse event was similar in the icosapent ethyl group compared with the placebo group irrespective of SMuRF burden, including SMuRF-less: (46/61 [75.4 %] vs 53/70 [75.7 %]), one SMuRF (510/668 [76.3 %] vs 468/635 [73.7 %]), two (931/1169 [79.6 %] vs 970/1211 [80.1 %]), three (719/827 [86.9 %] vs 710/815 [87.1 %]), and four (154/167 [92.2 %] vs 149/159 [93.7 %]).

## 4 Discussion

In this analysis, among statin-treated patients with elevated triglycerides, established CVD, and controlled LDL-C, greater SMuRF burden was associated with increased CV event rates in the placebo group. There was no evidence of treatment heterogeneity by baseline SMuRF burden, suggesting icosapent ethyl reduced CV events across these different risk profiles. Although point estimates suggested benefit among SMuRF-less patients, the small sample size limited precision.

Icosapent ethyl targets residual CV risk not addressed by traditional SMuRF therapy, which may explain its benefit across different SMuRF burden. While SMuRFs are appropriately the focus of traditional preventive strategies, it is important to recognize and appropriately treat patients who may have residual risk beyond SMuRFs. SMuRF-less patients remain at meaningful CV risk, as demonstrated by the event rates observed in this analysis. Prior data have suggested that SMURF-less patients with myocardial infarctions are at higher risk for mortality than their counterparts with SMURFs [4]. Additionally, because of the lack of commonly recognized risk factors, these patients are often underrepresented or understudied in clinical trials [1].

Possible pathways for residual risk include inflammation, triglyceride-rich lipoproteins and their remnants, platelet activation and thrombosis, endothelial dysfunction, and plaque instability [[5], [6], [7], [8]]. Icosapent ethyl has multiple biological effects, including anti-inflammatory properties, anti-platelet effects, membrane lipid stabilization, and reduction in lipid oxidation, all pathways which may reduce CV risk in addition to traditional SMuRFs [5,8,9]. Further study of these pathways and the effect of icosapent ethyl on them could lead to meaningful insight on residual CV risk.

We acknowledge the limitations of this analysis. This post hoc analysis was not stratified by SMuRF burden and involved multiple subgroup comparisons. Because of the possibility of Type I error, the findings should be considered hypothesis generating. The small number of SMuRF-less patients limited precision of effect estimates, including the NNTs, and thus these findings should be interpreted as exploratory. Residual confounding across SMuRF strata cannot be excluded. Generalizability is restricted to statin-treated patients with elevated triglycerides and established CVD or diabetes enrolled in REDUCE-IT. Additionally, some of the SMuRF-less patients could have had risk factors that were undiagnosed, though this is less likely in clinical trial populations compared with registries [10].

In conclusion, icosapent ethyl reduced the rate of CV events among statin-treated patients with established CVD, elevated triglycerides, and controlled LDL-C, across the range of SMuRF burden. These findings support wider use of icosapent ethyl as an effective statin adjunct to reduce CV events in eligible patients.

## Funding information

REDUCE-IT was funded by Amarin, as were these analyses. Dr. Aggarwal receives research training support from the National Heart, Lung, and Blood Institute grant 5T32HL007604.

## CRediT authorship contribution statement

**Rahul Aggarwal:** Writing – review & editing, Writing – original draft, Methodology, Investigation, Conceptualization. **Deepak L. Bhatt:** Writing – review & editing, Supervision, Methodology, Investigation, Conceptualization. **Michael Miller:** Writing – review & editing, Investigation. **Christie M. Ballantyne:** Writing – review & editing, Investigation. **Eliot A. Brinton:** Writing – review & editing, Investigation. **Terry A. Jacobson:** Writing – review & editing, Investigation. **Steven B. Ketchum:** Writing – review & editing, Investigation. **Jean-Claude Tardif:** Writing – review & editing, Investigation. **Matthew J. Budoff:** Writing – review & editing, Investigation. **Gemma A. Figtree:** Writing – review & editing, Investigation. **Ph․ Gabriel Steg:** Writing – review & editing, Investigation.

## Declaration of competing interest

The authors declare the following financial interests/personal relationships which may be considered as potential competing interests: Dr. Aggarwal is involved in research funded by the Bristol Myers Squibb-Pfizer alliance, Novartis, Lexicon, Cleerly, and Amarin, is a consultant for Lexicon, Bayer, and Amarin, and has served on an advisory board for Bayer. Dr. Bhatt served as the Chair of the REDUCE-IT Steering Committee with research funding paid to Brigham and Women’s Hospital and Icahn School of Medicine at Mount Sinai and discloses the following relationships - Advisory Board: Angiowave, Antlia Bioscience, Bayer, Boehringer Ingelheim, CellProthera, Cereno Scientific, E-Star Biotech, High Enroll, Janssen, Level Ex, McKinsey, Medscape Cardiology, Merck, NirvaMed, Novo Nordisk, Repair Biotechnologies, Stasys, SandboxAQ (stock options), Tourmaline Bio; Board of Directors: American Heart Association New York City, Angiowave (stock options), Bristol Myers Squibb (stock), DRS.LINQ (stock options), High Enroll (stock); Consultant: Alnylam, Altimmune, Broadview Ventures, Corcept Therapeutics, Corsera, GlaxoSmithKline, Hims, SERB, SFJ, Summa Therapeutics, Worldwide Clinical Trials; Data Monitoring Committees: Acesion Pharma, Assistance Publique-Hôpitaux de Paris, Baim Institute for Clinical Research, Boston Scientific (Chair, PEITHO trial), Cleveland Clinic, Contego Medical (Chair, PERFORMANCE 2), Duke Clinical Research Institute, Mayo Clinic, Mount Sinai School of Medicine (for the ABILITY-DM trial, funded by Concept Medical; for ALLAY-HF, funded by Alleviant Medical), Novartis, Population Health Research Institute; Rutgers University (for the NIH-funded MINT Trial); : American College of Cardiology (Senior Associate Editor, Clinical Trials and News, ACC.org; Chair, ACC Accreditation Oversight Committee), Arnold and Porter law firm (work related to Sanofi/Bristol-Myers Squibb clopidogrel litigation), Baim Institute for Clinical Research (AEGIS-II executive committee funded by CSL Behring), Belvoir Publications (Editor in Chief, Harvard Heart Letter), Canadian Medical and Surgical Knowledge Translation Research Group (clinical trial steering committees), CSL Behring (AHA lecture), Duke Clinical Research Institute, Engage Health Media, HMP Global (Editor in Chief, Journal of Invasive Cardiology), Medtelligence/ReachMD (CME steering committees), MJH Life Sciences, Oakstone CME (Course Director, Comprehensive Review of Interventional Cardiology), Philips (Becker's Webinar on AI), 10.13039/100030936Population Health Research Institute, WebMD (CME steering committees), 10.13039/100005134Wiley (steering committee); Other: Clinical Cardiology (Deputy Editor, unpaid); Progress in Cardiovascular Diseases (Deputy Editor, unpaid); Added Health (Editorial Board; stock options); Patent: Sotagliflozin (named on a patent for sotagliflozin assigned to Brigham and Women's Hospital who assigned to Lexicon; neither I nor Brigham and Women's Hospital receive any income from this patent); Research Funding: 10.13039/100009014Abbott, Acesion Pharma, Afimmune, 10.13039/100006400Alnylam, 10.13039/100014389Amarin, 10.13039/100016579Amgen, 10.13039/100004325AstraZeneca, 10.13039/100007330Atricure, 10.13039/100004326Bayer, 10.13039/100008349Boehringer Ingelheim, 10.13039/100008497Boston Scientific, CellProthera, Cereno Scientific, 10.13039/100007560Chiesi, Cleerly, 10.13039/100008322CSL Behring, Faraday Pharmaceuticals, Fractyl, 10.13039/501100016198Idorsia, 10.13039/100014554Janssen, 10.13039/501100003490Javelin, Lexicon, 10.13039/100019518Lilly, 10.13039/100004374Medtronic, 10.13039/100004334Merck, MiRUS, 10.13039/100019533Moderna, 10.13039/100014444Novartis, 10.13039/501100004191Novo Nordisk, 10.13039/100004319Pfizer, PhaseBio, 10.13039/100009857Regeneron, Reid Hoffman Foundation, 10.13039/501100019579Roche, 10.13039/100004339Sanofi, Stasys, 89Bio; Royalties: Elsevier (Editor, Braunwald’s Heart Disease); Site Co-Investigator: Cleerly. Dr. Miller reports serving as a scientific advisor (Amarin, Ionis, 89bio, New Amsterdam) and in a DSMB Committee (dalCor). Dr. Ballantyne has received grant/research support (through his institution) from 10.13039/100014386Abbott Diagnostic, Akcea, 10.13039/100002429Amgen, 10.13039/100021688Arrowhead, 10.13039/100020536Eli Lilly, 10.13039/100013669Ionis, 10.13039/100004334Merck, New Amsterdam, 10.13039/100004336Novartis, 10.13039/501100009708Novo Nordisk, and 10.13039/100016545Roche Diagnostic for contracted research and consulting fees from 89Bio, Abbott Diagnostics, Amgen, Arrowhead, Astra Zeneca, Denka Seiken, Esperion, Genentech, Ionis, Eli Lilly, Merck, New Amsterdam, Novartis, Novo Nordisk, and Roche Diagnostic as a consultant. Dr Brinton has received speaker fees from Amarin, Amgen, Amryt, and Esperion; and has received consulting fees from 89Bio, Amarin, Amgen, Amryt, DelCor, Esperion, Immunovant, Ionis, Merck, NovoNordisk, and Pfizer. Dr. Ketchum is an employee and stockholder in Amarin Pharma, Inc. Dr. Tardif reports research grants from 10.13039/100014389Amarin, Boehringer-Ingelheim, Ceapro, 10.13039/501100016186DalCor Pharmaceuticals, 10.13039/100004334Merck, 10.13039/100004336Novartis, Novo-Nordisk, 10.13039/100004319Pfizer, and Verve Therapeutics; honoraria from 10.13039/501100016186DalCor Pharmaceuticals and 10.13039/100004319Pfizer; minor equity interest from 10.13039/501100016186DalCor Pharmaceuticals; and authorship of patents on pharmacogenomics-guided CETP inhibition and use of colchicine after myocardial infarction. Dr. Figtree reports personal consulting fees from AstraZeneca, CSL Ltd, Amgen, and Janssen, and grants from 10.13039/100004339Sanofi outside the submitted work. Dr Steg has received research grants from 10.13039/100014389Amarin and 10.13039/100004339Sano; has served on clinical trials (steering committee, clinical events committee, data and safety monitoring board [DSMB]) for Amarin, Amgen, AstraZeneca, Bayer, Bristol Myers Squibb, CSL Behring, Idorsia, Janssen, Novartis, Novo Nordisk, Pfizer, and Sano; has served as a consultant or speaker for Amarin, Amgen, BMS, and Novo Nordisk; and is Senior Associate Editor at Circulation.

## References

[1] Figtree G.A., Vernon S.T., Harmer J.A. (2023). Clinical pathway for coronary atherosclerosis in patients without conventional modifiable risk factors: JACC State-of-the-art review. J Am Coll Cardiol.

[2] Bhatt D.L., Steg P.G., Miller M. (2019). Cardiovascular risk reduction with icosapent ethyl for hypertriglyceridemia. N Engl J Med.

[3] Bhatt D.L., Steg P.G., Brinton E.A. (2017). Rationale and design of REDUCE-IT: reduction of cardiovascular events with icosapent ethyl-intervention trial. Clin Cardiol.

[4] Figtree G.A., Vernon S.T., Hadziosmanovic N. (2021). Mortality in STEMI patients without standard modifiable risk factors: a sex-disaggregated analysis of SWEDEHEART registry data. Lancet Lond Engl.

[5] Sherratt S.C.R., Libby P., Bhatt D.L., Mason R.P. (2022). A biological rationale for the disparate effects of omega-3 fatty acids on cardiovascular disease outcomes. Prostaglandins Leukot Essent Fatty Acids.

[6] Sherratt S.C.R., Libby P., Budoff M.J., Bhatt D.L., Mason R.P. (2023). Role of omega-3 fatty acids in cardiovascular disease: the debate continues. Curr Atheroscler Rep.

[7] Bhatt D.L., Libby P., Mason R.P. (2025). Emerging pathways of action of eicosapentaenoic acid (EPA). JACC Basic Transl Sci.

[8] Mourikis P., Benkhoff M., Wildeis L. (2025). Icosapent ethyl reduces arterial thrombosis by inhibition of cyclooxygenase-1-induced platelet reactivity. Sci Transl Med.

[9] Mason R.P., Libby P., Bhatt D.L. (2020). Emerging mechanisms of cardiovascular protection for the omega-3 fatty acid eicosapentaenoic acid. Arterioscler Thromb Vasc Biol.

[10] Nurmohamed N.S., Ngo-Metzger Q., Taub P.R. (2025). First myocardial infarction: risk factors, symptoms, and medical therapy. Eur Heart J.

